# Supplementary material for: Transposon silencing in the Drosophila female germline is essential for genome stability in progeny embryos
Source: Life Sci Alliance. 2018 Sep 17;1(5):e201800179. doi: 10.26508/lsa.201800179 (PMC6238532; doi:10.26508/lsa.201800179)
Supplement: Supplementary file 1 [file LSA-2018-00179_TableS1.docx]

Supplementary Table S1 (related to Fig 1): PGC-positive embryos (3 replicates).

| Genotypes | # of PGC positive embryos | Total # of embryos |
| --- | --- | --- |
| *w^1118^* | 85 | 85 |
| *vas^D1^/vas^D1^;GFP-vas^wt^/nos-Gal4* | 19 | 52 |
| *vas^D1^/vas^D1^;GFP-vas^wt^/vas-Gal4* | 29 | 82 |
|  | | |
| *w^1118^* | 89 | 89 |
| *vas^D1^/vas^D1^;GFP-vas^wt^/nos-Gal4* | 22 | 42 |
| *vas^D1^/vas^D1^;GFP-vas^wt^/vas-Gal4* | 29 | 68 |
|  | | |
| *w^1118^* | 68 | 68 |
| *vas^D1^/vas^D1^;GFP-vas^wt^/nos-Gal4* | 13 | 57 |
| *vas^D1^/vas^D1^;GFP-vas^wt^/vas-Gal4* | 45 | 77 |
