# Supplementary material for: Transposon silencing in the Drosophila female germline is essential for genome stability in progeny embryos
Source: Life Sci Alliance. 2018 Sep 17;1(5):e201800179. doi: 10.26508/lsa.201800179 (PMC6238532; doi:10.26508/lsa.201800179)
Supplement: Supplementary file 2 [file LSA-2018-00179_TableS2.docx]

Supplementary Table S2 (related to Fig 1 and supplementary Fig S1): Egg-laying and hatching rates (3 replicates).

| Genotypes/Crosses | Average # of eggs laid per female per day | Average # of hatched larvae |
| --- | --- | --- |
| *w^1118^* | 54 | 54 |
| *vas^D1^/vas^D1^;GFP-vas^WT^/nos-Gal4* | 35 | 7 |
| *vas^D1^/vas^D1^;GFP-vas^WT^/vas-Gal4* | 52 | 18 |
|  | | |
| *w^1118^* | 51 | 51 |
| *vas^D1^/vas^D1^;GFP-vas^WT^/nos-Gal4* | 39 | 5 |
| *vas^D1^/vas^D1^;GFP-vas^WT^/vas-Gal4* | 38 | 13 |
|  | | |
| *w^1118^* | 59 | 58 |
| *vas^D1^/vas^D1^;GFP-vas^WT^/nos-Gal4* | 44 | 9 |
| *vas^D1^/vas^D1^;GFP-vas^WT^/vas-Gal4* | 40 | 16 |
