# Supplementary material for: Transposon silencing in the Drosophila female germline is essential for genome stability in progeny embryos
Source: Life Sci Alliance. 2018 Sep 17;1(5):e201800179. doi: 10.26508/lsa.201800179 (PMC6238532; doi:10.26508/lsa.201800179)
Supplement: Supplementary file 3 [file LSA-2018-00179_TableS3.docx]

Supplementary Table S3 (Supplementary Fig S2): Egg-laying and hatching rates (3 replicates).

| Genotypes/Crosses | Average # of eggs laid per female per day | Average # of hatched larvae |
| --- | --- | --- |
| *w^1118^* | 48 | 48 |
| *vas^D1^/vas^Q7^;GFP-vas^WT^/nos-Gal4* | 44 | 8 |
|  | | |
| *w^1118^* | 50 | 47 |
| *vas^D1^/vas^Q7^;GFP-vas^WT^/nos-Gal4* | 36 | 6 |
|  | | |
| *w^1118^* | 51 | 50 |
| *vas^D1^/vas^Q7^;GFP-vas^WT^/nos-Gal4* | 42 | 10 |
