# Supplementary material for: Transposon silencing in the Drosophila female germline is essential for genome stability in progeny embryos
Source: Life Sci Alliance. 2018 Sep 17;1(5):e201800179. doi: 10.26508/lsa.201800179 (PMC6238532; doi:10.26508/lsa.201800179)
Supplement: Supplementary file 4 [file LSA-2018-00179_TableS4.docx]

Supplementary Table S4 (related to Fig 2): Nuclear damage occurrence in embryos (3 replicates).

| Genotypes | # of embryos with nuclear damage | Total # of embryos |
| --- | --- | --- |
| *w^1118^* | 0 | 85 |
| *vas^D1^/vas^D1^;GFP-vas^WT^/nos-Gal4* | 42 | 94 |
| *vas^D1^/vas^D1^;GFP-vas^WT^/vas-Gal4* | 9 | 91 |
|  | | |
| *w^1118^* | 2 | 92 |
| *vas^D1^/vas^D1^;GFP-vas^WT^/nos-Gal4* | 18 | 61 |
| *vas^D1^/vas^D1^;GFP-vas^WT^/vas-Gal4* | 4 | 72 |
|  | | |
| *w^1118^* | 1 | 69 |
| *vas^D1^/vas^D1^;GFP-vas^WT^/nos-Gal4* | 22 | 79 |
| *vas^D1^/vas^D1^;GFP-vas^WT^/vas-Gal4* | 4 | 81 |
