# Supplementary material for: Transposon silencing in the Drosophila female germline is essential for genome stability in progeny embryos
Source: Life Sci Alliance. 2018 Sep 17;1(5):e201800179. doi: 10.26508/lsa.201800179 (PMC6238532; doi:10.26508/lsa.201800179)
Supplement: Supplementary file 5 [file LSA-2018-00179_TableS5.docx]

Supplementary Table S5 (related to supplementary Fig S4): Egg-laying and hatching rates (3 replicates).

| Genotypes/Crosses | Average # of eggs laid per female | Average # of hatched larvae |
| --- | --- | --- |
| *w^1118^* | 54 | 53 |
| *vas^D1^, mnk^P6^/*  *vas^D1^, mnk^P6^* | 20 | 0 |
| *mnk^P6^/ mnk^P6^* | 51 | 31 |
| *ago^t2^/ ago^t3^* | 5 | 0 |
|  | | |
| *w^1118^* | 51 | 50 |
| *vas^D1^, mnk^P6^/*  *vas^D1^, mnk^P6^* | 28 | 0 |
| *mnk^P6^/ mnk^P6^* | 50 | 24 |
| *ago^t2^/ ago^t3^* | 3 | 0 |
|  | | |
| *w^1118^* | 51 | 51 |
| *vas^D1^, mnk^P6^/*  *vas^D1^, mnk^P6^* | 22 | 0 |
| *mnk^P6^/ mnk^P6^* | 45 | 19 |
| *ago^t2^/ ago^t3^* | 5 | 0 |
