# Supplementary material for: Transposon silencing in the Drosophila female germline is essential for genome stability in progeny embryos
Source: Life Sci Alliance. 2018 Sep 17;1(5):e201800179. doi: 10.26508/lsa.201800179 (PMC6238532; doi:10.26508/lsa.201800179)
Supplement: Supplementary file 6 [file LSA-2018-00179_TableS6.docx]

Supplementary Table S6: Recombination and crossing scheme for generation of *mnk, vas* double mutant flies. (Numbers in parentheses represent likelihood of recombination according to a genomic distance of 4cM between *mnk* and *vas*).

P:

♀

*+, +, mnk^P6[P{lacW}]^*

*CyO*

X

*b^1^, vas^D1^, + CyO*

♂

F1:

♀

*+, +, mnk^P6[P{lacW}]^*

*b^1^, vas^D1^, +*

X

*CyOif*

♂

F2:

*+, +, mnk^P6[P{lacW}]^*

*CyO*

*+, +, + CyO*

*b^1^, vas^D1^, + CyO*

*b^1^, vas^D1^, mnk^P6[P{lacW}]^*

*CyO*

[2%]

[2%]

[48%]

[48%]

F3:

red

red

white

white

*+, +, mnk^P6[P{lacW}]^*

*+, +, mnk^P6[P{lacW}]^*

*b^1^, vas^D1^, mnk^P6[P{lacW}]^*

*b^1^, vas^D1^, mnk^P6[P{lacW}]^*

bright

dark

1^st^ screen:

eye color

2^nd^ screen:

body color

X

*CyO*

*if*

X

*CyO*

*if*

3 positive stocks

200

single crosses
