# Supplementary material for: Transposon silencing in the Drosophila female germline is essential for genome stability in progeny embryos
Source: Life Sci Alliance. 2018 Sep 17;1(5):e201800179. doi: 10.26508/lsa.201800179 (PMC6238532; doi:10.26508/lsa.201800179)
Supplement: Supplementary file 7 [file LSA-2018-00179_TableS7.docx]

Supplementary Table S7: Sequences of primers used for qPCR and DNA oligonucleotides used for FISH

| rp49_fwd | ATGACCATCCGCCCAGCATAC |
| --- | --- |
| rp49_rev | CTGCATGAGCAGGACCTCCAG |
| 18S rRNA_fwd | CGGAGAGGGAGCCTGAGAA |
| 18S rRNA_rev | CCAATTGGTCCTTGTTAAAG |
| burdock_fwd | AGGGAAATATTTGGCCATCC |
| burdock_rev | TTTTGGCCCTGTAAACCTTG |
| blood_fwd | CCAACAAAGAGGCAAGACCG |
| blood_rev | TCGAGCTGCTTACGCATACTGTC |
| HeT-A_fwd | GCTTCAGGCATGCCAAAAACTC |
| HeT-A_rev | GTACGCGCTAATATGCTGCC |
| gypsy_fwd | CTTCACGTTCTGCGAGCGGTCT |
| gypsy_rev | CGCTCGAAGGTTACCAGGTAGGTTC |
| mnk_fwd | GAAGAAACGCTCAGAGATTC |
| mnk_rev | CAACAGAAAAGGACGAATGC |
| HeT-A FISH probe | TTTTTAGCACGTCCGCACG |
| HeT-A FISH probe | TTGCTCAGGATTGCTGAGC |
| HeT-A FISH probe | TTACTTGTGTGTCGGCAGC |
| HeT-A FISH probe | TGTGGACGGATTTTGCAGC |
| HeT-A FISH probe | TGTCAACGTTGTTTGCCGC |
| HeT-A FISH probe | TGGGTCAGCGATTTAGCTG |
| HeT-A FISH probe | TGGCGTGGTATCGTCAATG |
| HeT-A FISH probe | TGCCCTTCTCAGATTCCTG |
| HeT-A FISH probe | TGCAACAGGAAGACAGGTG |
| HeT-A FISH probe | TCCAGATGTCTTTGCGCTG |
| HeT-A FISH probe | TCAGCTCCTCGCTAGTGA |
| HeT-A FISH probe | TCAGACGCTTCTGAATGGC |
| HeT-A FISH probe | TACTTGTCCTATGTGCCGG |
| HeT-A FISH probe | TAAAAGCAGACCGACGTGC |
| HeT-A FISH probe | GTGCATAAGTTGCTGCAGC |
| HeT-A FISH probe | GTGACAGAGGAGTCGTCA |
| HeT-A FISH probe | GTCGTCAGGAAGAGGGAA |
| HeT-A FISH probe | GTCAATGCAGTGGCATCAG |
| HeT-A FISH probe | GGTGGTGTCTTCTGTCTCA |
| HeT-A FISH probe | GGGATACTACAGGAGATCG |
| HeT-A FISH probe | GAAGGTGATGACGGTGAAG |
| HeT-A FISH probe | CTTGGCTTAAAGCTGGCTC |
| HeT-A FISH probe | CTCTTCTACCCTCATCGG |
| HeT-A FISH probe | CGTCCAAGAGGCCTTTTTG |
| HeT-A FISH probe | CCCAGAGCAATTTACGCAG |
| HeT-A FISH probe | CAGTAGGATGGAGCTGCA |
| HeT-A FISH probe | CAGGTACGTTTGCTTGGAG |
| HeT-A FISH probe | AGTTGTGTACTTGGGCTGG |
| HeT-A FISH probe | ACTTTGCTGGTGGAGGTAC |
| HeT-A FISH probe | AAGGAGTTGCGTGGTTGTC |
| HeT-A FISH probe | TTTTGGCATGCCTGAAGCC |
| HeT-A FISH probe | TTTGGCCATGACGATCTCC |
| HeT-A FISH probe | TTTATAGAGCGTGCGTCCG |
| HeT-A FISH probe | TTCTGACGAATCGCGCTTG |
| HeT-A FISH probe | TTCCTCTTGCTTGCGTTCG |
| HeT-A FISH probe | TGGAGTGGTGGAGATGTC |
| HeT-A FISH probe | TGCTAGTGTGAGTGTGTGC |
| HeT-A FISH probe | TGATGACTCGGAAGCCTC |
| HeT-A FISH probe | TCAATGTCCACCCTTTGCC |
| HeT-A FISH probe | TCAAACATTCGCATGGGGC |
| HeT-A FISH probe | GTTATAGGCGGTCATGTCC |
| HeT-A FISH probe | GTCCAGATCGTCGTTTGTC |
| HeT-A FISH probe | GGGCGTCTTAAAGTTGGAG |
| HeT-A FISH probe | GCTTCGGGAGGATGATGA |
| HeT-A FISH probe | GCTCTTGAAAACGGGAGTG |
| HeT-A FISH probe | GCTCCCGTGTCCTGTTTT |
| HeT-A FISH probe | GCGCTCTTTTTATGAGGGG |
| HeT-A FISH probe | GCCCTAGTAGTATAGCTGG |
| HeT-A FISH probe | CTCCTGCTGTGTAGTTCAG |
| HeT-A FISH probe | ATTCACCACAGTGGGCTTG |
| HeT-A FISH probe | ATCCGAGCTTCAGCAGTTC |
| HeT-A FISH probe | ATAAATCCCGCTTGGCTGC |
| HeT-A FISH probe | AGAGAGAGGGGAAACTTCC |
| HeT-A FISH probe | ACTCATAGGCTGCTCGTC |
| HeT-A FISH probe | AATCATCCTGAGCGGAAGG |
| HeT-A FISH probe | AAAGGAAGTCCGTTGGCCA |
| HeT-A FISH probe | TATAGCAGCCCCAGAAGAG |
| HeT-A FISH probe | GTCTGCTTGATTTGAGGGC |
| HeT-A FISH probe | CGGAGAAGATCGCTGTTC |
| HeT-A FISH probe | TGTTTATTGTTGCCGCGGC |
| HeT-A FISH probe | TTCTTTGCAGCCTGAGGAC |
| HeT-A FISH probe | AGTTATGCGCGTGAGAGTC |
| HeT-A FISH probe | CGTCGCGGTTCAAATTTTGC |
| HeT-A FISH probe | GCGCGTGGAGTATTATGTAG |
| HeT-A FISH probe | GCTATGCTGGTGGATTTAGC |
| HeT-A FISH probe | CTGAGAATTGTCTGATCCGTG |
| HeT-A FISH probe | CCCTGGCTTTCTTTAATTGGG |
| HeT-A FISH probe | CTGGTTGCTTTCTTCTCTTCG |
| HeT-A FISH probe | CAAATGTTCGCTTTTCGCGTG |
| HeT-A FISH probe | GCAGCTTGTCGGTTTGCAC |
| HeT-A FISH probe | CATGGGCGATATATTGAGGTAG |
| HeT-A FISH probe | CTTCGTCTCCGTTTTGTTATGG |
| burdock FISH probe | TCAAGCCAAACGGCAAACG |
| burdock FISH probe | CGCTCTATCCTTTTCTGCG |
| burdock FISH probe | TATCGACTACCCAAACCGG |
| burdock FISH probe | GTTGTAAGGTGACGACGAG |
| burdock FISH probe | CACAAAATCCGATACGCCC |
| burdock FISH probe | AAATGGGTTGGTCGTCCTC |
| burdock FISH probe | ATACGGCAGTGCTTCTTCC |
| burdock FISH probe | TTCCGCAAAGACGGCCAA |
| burdock FISH probe | TGGTTCTCCCTGCTATCTC |
| burdock FISH probe | TCTATGTACGGTAGCAGGG |
| burdock FISH probe | TCATCAACCTCCGCTTCTG |
| burdock FISH probe | CTGCCACTCGTTGGTCTT |
| burdock FISH probe | GATGCTTGCGCATACCCAA |
| burdock FISH probe | TTTTGCTGTTGGGGTTGCG |
| burdock FISH probe | AAGGGCGAATTGGTAACGC |
| burdock FISH probe | ATGGTTCGACTCGACCTC |
| burdock FISH probe | CGCTAAAGCGAGAGCAGT |
| burdock FISH probe | GTTTTGTCTCCGGATCCTC |
| burdock FISH probe | TTACAATAGGCTTGCGCGG |
| burdock FISH probe | AGCACTTGAGGCTTACGAC |
| burdock FISH probe | CAGAATTTGGTGAAACGCCG |
| burdock FISH probe | GGGGTACGGATAGAGTTTTG |
| burdock FISH probe | GTAATTTTTCGAAGCCCCGG |
| burdock FISH probe | GGGATGGCCAAATATTTCCC |
| burdock FISH probe | GTCGGAAATGGAGTTTGTCC |
| burdock FISH probe | GTTCGATTTTCTGACCCCTG |
| burdock FISH probe | GCTCCGGTTACAAATACACG |
| burdock FISH probe | CATTCAGTGACATCGCCAAG |
| burdock FISH probe | CAGTAAGAGTCATGTCTCCC |
| burdock FISH probe | GCCATCAAAATCTGGCAGAG |
| burdock FISH probe | TTACAATAGGCTTGCGCGG |
| burdock FISH probe | AGCACTTGAGGCTTACGAC |
| burdock FISH probe | TCTAAGACGAAGGCTGTCC |
| burdock FISH probe | TGAAAGCGGATCTTACGGG |
| burdock FISH probe | TACTGCCAACCTGGTACTG |
| burdock FISH probe | AAACCATCGAAGGGAAGGC |
| burdock FISH probe | CTCTGCGTGTCTGGATGA |
| burdock FISH probe | TCTCCATGTCATGGGTTCG |
| burdock FISH probe | TCCCTCTTCTACTCTTCTC |
| burdock FISH probe | GAGTGAGTGCGCTATACTG |
| burdock FISH probe | TGCTAACTTGGCGATGGTC |
| burdock FISH probe | TTATGAGGACCGTTGTCCC |
| burdock FISH probe | CCTCAAAGGTTCTGTCTCG |
| burdock FISH probe | TATGCGCTCTGTTGTGCTC |
| burdock FISH probe | TCGGAAGGTTGTTGCTGGA |
| burdock FISH probe | TCGCATTCACTACATCCGG |
| burdock FISH probe | GATGCCTTGTCTTGCTTCC |
| burdock FISH probe | ACAGTTAACCGGCTTGTCG |
| burdock FISH probe | GTTGCAATGTCTGACTGGG |
| burdock FISH probe | ACATAGGTCTCCTTGCCAG |
| burdock FISH probe | TGTCAGTGAGTGGTCTAGC |
| burdock FISH probe | GAAACCCTCATGTTTGCCC |
| burdock FISH probe | GACAGTCTGTCTAGTACCC |
| burdock FISH probe | GCAACGTCGTTTACGTGG |
| burdock FISH probe | GACCGACGCTTCTAATCTTC |
| burdock FISH probe | CAAGGTTCTGGAGATGATCG |
| burdock FISH probe | GTAACCGTCGACTTGTTGAC |
| burdock FISH probe | CGTATGTGTTGAACGCACTC |
| burdock FISH probe | CAGTTCGTCGTTTCAGTACC |
| burdock FISH probe | GCTGTTTTTCCTGAGCTTCG |
